# When Fussiness Is Not Just Fussiness: A Case Report of Early Wolff-Parkinson-White Syndrome Recognized in Primary Care

**DOI:** 10.7759/cureus.108382

**Published:** 2026-05-06

**Authors:** Richard Nguyen, Vanikaa Keswani, Katie P Nguyen

**Affiliations:** 1 Family Medicine, University of California, San Francisco (UCSF) Fresno, Fresno, USA; 2 Pediatrics, University of California, San Francisco (UCSF) Fresno, Fresno, USA

**Keywords:** antiarrhythmic medication therapy, electrocardiogram (ecg/ekg), supraventricular tachycardia (svt), term neonate, wolff-parkinson-white (wpw) syndrome

## Abstract

This case report describes a two-week-old term infant who initially presented with nonspecific symptoms of vomiting and diarrhea in the outpatient setting. Despite an unremarkable birth history, the patient’s clinical condition deteriorated, and subsequent evaluation in the emergency department revealed supraventricular tachycardia (SVT), necessitating multiple drug and direct-current cardioversion attempts. The infant was promptly transferred to a tertiary care center with a pediatric intensive care unit, cardiology, and electrophysiology. Initial electrocardiogram showed a wide complex tachycardia concerning for SVT, later revealing Wolff-Parkinson-White syndrome, and started on antiarrhythmic therapy. This report highlights the critical role of primary care providers in recognizing and managing early-onset SVT to prevent acute heart failure and other morbidity and mortality.

## Introduction

Wolff-Parkinson-White (WPW) syndrome is a congenital cardiac pre-excitation syndrome characterized by an accessory pathway (bundle of Kent) between the atria and ventricles, leading to premature excitation of the ventricles. The most common arrhythmia seen with WPW syndrome is supraventricular tachycardia (SVT), which, if left untreated, can be life-threatening. SVT is also the most common arrhythmia for neonates and infants [1]. The incidence of WPW syndrome is 0.9-3% in the general population [2]. The prevalence of WPW can vary from one in 25,000 to one in 250 children [1]. Specifically, the estimated neonatal incidence for WPW of 0.1% to 0.2% may be misrepresented secondary to missed or delayed diagnosis [3]. WPW can present with nonspecific symptoms such as intermittent tachycardia, poor feeding, or unexplained weight loss. With clinical acumen, this is where the primary care provider (PCP) or pediatric emergency physician (Peds EM) can have the most immediate impact in the patient’s course with prompt recognition and management.

The first WPW onset is most frequent in children under one year old, but studies suggest two peak onsets first during infancy and a second between eight and 12 years old [2]. There is an incidence rate of 35% for congestive heart failure in infants under four months of age if there is a delay in treatment [1]. The hallmark electrocardiographic (ECG) findings for the WPW pattern consist of a short PR interval and a prolonged QRS complex with an initial slurring upstroke called the delta wave, noted during sinus rhythm. The difference between the WPW pattern and WPW syndrome lies in the absence or presence of symptomatic arrhythmias. It is challenging to accurately determine the prevalence of the WPW pattern because these individuals have no clinical symptoms and are only found incidentally. WPW syndrome occurs when pre-excitation leads to both ECG findings and the symptomatic arrhythmia [4]. Clinical manifestations range from asymptomatic presentation (WPW pattern) to paroxysmal SVT, syncope, or, in rare cases, sudden cardiac death. While most cases of SVT are not associated with congenital heart disease, WPW has been linked to structural abnormalities, most commonly Ebstein’s anomaly [3]. Current recommendations on management of WPW in prenatal and postnatal periods are based on standards of care for the adult population. There is a paucity of evidence-based literature specifically on the true incidence and long-term implications for neonates with WPW [3]. Given the potential for missed or delayed diagnoses, it is imperative that PCP and Peds EM recognize early yet subtle warning signs (tachycardia, poor feeding, unexplained weight loss) and conduct a thorough evaluation to facilitate timely intervention.

## Case presentation

Outpatient care

At 14 days of life (DOL), a previously healthy female infant presented to her PCP with two days of vomiting and diarrhea. She was born at term via an uncomplicated vaginal delivery. There was no family history of congenital heart defect, sudden death, or arrhythmias. She passed her routine newborn screening tests, including the congenital heart disease screen. She was discharged at DOL 2 without complications. Her birth weight was 2.85 kilograms (kg); outpatient records indicated the initial expected newborn weight loss to 2.72 kg with a regain in weight to 3.37 kg (Table 1). Her vital signs were stable, and the physical exam was unremarkable. The parent was advised to closely monitor the patient and to return if there are further concerns with feeding, trouble breathing, skin changes, or a change in activity level. Clinic records indicate the patient had four visits with varying providers for similar concerns. On DOL 18, the parents brought the patient back for reevaluation. The patient was then directed to the emergency department (ED) for evaluation of persistent feeding difficulties, new weight loss, and increased work of breathing.

**Table 1:** Summary of vital signs, electrocardiogram, and echocardiogram DOL: days of life; ED: emergency department; PICU: pediatric intensive care unit; HR: heart rate; RR: respiratory rate; ECG: electrocardiogram; VT: ventricular tachycardia; SVT: supraventricular tachycardia; WPW: Wolff-Parkinson-White.

	HR	RR	Oxygen saturation	Weight	ECG	ECHO
Birth	160	52	N/A	2.85 kg	N/A	N/A
DOL 2	171	36	96%	2.72 kg	N/A	N/A
DOL 9	136	N/A	97%	3.37 kg	N/A	N/A
DOL 14	136	36	99%	3.25 kg	N/A	N/A
DOL 18	130	42	97%	3.2 kg	N/A	N/A
DOL 19 (ED)	263	60	92%	3.4 kg	Wide complex tachycardia at 262 bpm concerning for either SVT with aberrant conduction or VT	Ordered prior to transfer
20 DOL (PICU)	144	45	99%	3.53 kg	Sinus rhythm, evidence of ventricular pre-excitation consistent with WPW syndrome	Mildly diminished left and right ventricular systolic wall motion
39 DOL (Cardiology)	158	N/A	98%	4.1 kg	Sinus rhythm, evidence of ventricular preexcitation consistent with WPW pattern	Good ventricular function

Emergency department

**Figure 1:**
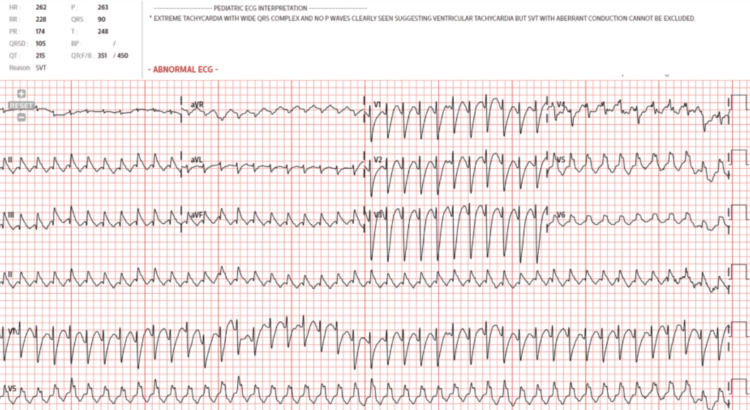
The initial electrocardiogram (ECG) showed a wide complex tachycardia at 262 bpm, concerning for either supraventricular tachycardia (SVT) with aberrant conduction or ventricular tachycardia (VT).

**Figure 2:**
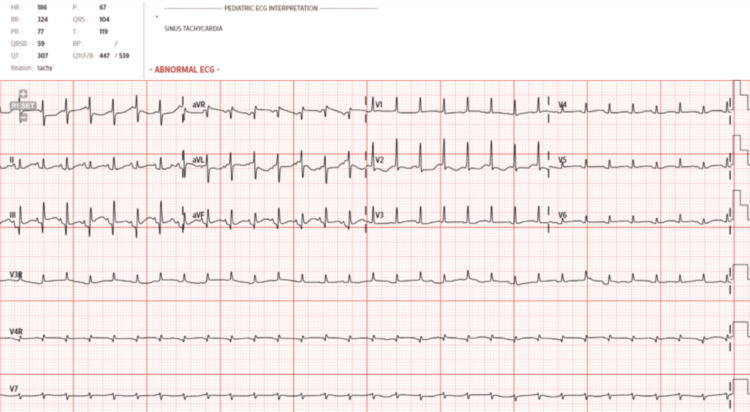
Repeat ECG showing sinus tachycardia with a heart rate of 186 bpm and borderline prolonged QTc after multiple drugs and direct-current cardioversion.

On arrival, the patient was afebrile, but systolic blood pressure was in the high 60s, with a heart rate (HR) of 263 bpm, respiratory rate of 60 breaths/min, and oxygen saturation of 92%. Physical exam showed a mottled infant with a weak cry, poor perfusion to the lower extremities, increased work of breathing, and inability to tolerate feeds. Arterial blood gas (ABG) showed a mixed respiratory and metabolic acidosis, in which she received a 20 cc/kg bolus of normal saline. The initial electrocardiogram (ECG) showed a wide complex tachycardia at 262 bpm, concerning for either SVT with aberrant conduction or ventricular tachycardia (VT) (Figure 1). Pediatric cardiology was consulted, who recommended vagal maneuvers first, then adenosine, and lastly direct-current cardioversion if needed. The vagal maneuvers (ice packs to the face) were unsuccessful. She received 1 mg/kg of and 2 mg/kg of adenosine with no change to the HR. She required cardioversion twice with 2 J/kg before returning to a sinus rhythm in the 180s bpm (Figure 2). An echocardiogram was ordered to evaluate cardiac function. The patient was promptly transferred to a higher tertiary care center with a pediatric intensive care unit (PICU).

PICU course

**Figure 3:**
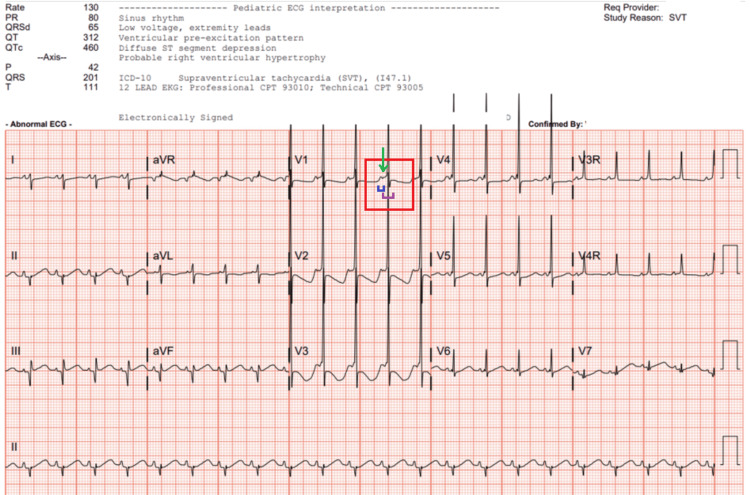
ECG showing sinus rhythm with a widened QRS complex with delta waves concerning for ventricular pre-excitation consistent with Wolff-Parkinson-White pattern. Delta wave (green), short PR interval (blue), and QRS widening (purple).

The patient required further cardioversion en route to the PICU. On arrival, ABG results showed a mixed respiratory and metabolic acidosis with a lactic acid level of 7.1, and she received 10 cc/kg of D10 (10% dextrose) bolus. The respiratory acidosis improved with a high-flow nasal cannula. Her ECG now demonstrated evidence of ventricular pre-excitation with delta waves consistent with WPW syndrome (Figure 3). She would continue to go back into SVT throughout her PICU course. She responded to adenosine but required escalation of the esmolol drip. She was then weaned off esmolol and transitioned to oral propranolol (4 mg/kg/day) as per electrophysiology (EP) recommendations. Her initial echocardiogram demonstrated depressed ventricular function, which improved at the time of discharge. The oral propranolol was decreased to 2 mg/kg/day, and oral amiodarone was started at 5 mg/kg/day with improved control for breakthrough SVT. She eventually stabilized with no further SVT episodes on the antiarrhythmic regimen (propranolol 2 mg/kg/day orally (PO), amiodarone 5 mg/kg/day PO, magnesium carbonate 10 mg/kg three times a day) and was cleared for discharge with close primary care and cardiology follow-up.

Outpatient cardiology

The patient is gaining weight and doing well. The 48-hour cardiac monitor confirmed no further episodes of SVT. She has been taking magnesium (used to stabilize the myocardium and prevent electrolyte imbalances), amiodarone, and propranolol as above. A repeat echocardiogram demonstrated normal segmental intracardiac anatomy with normal ventricular size and function. Her ECG showed ventricular pre-excitation with borderline prolonged QT and delta waves consistent with WPW. The plan is to wean the patient off amiodarone over three months. Amiodarone usage would require monitoring thyroid labs and liver function tests for the infant. A baseline ophthalmologic evaluation is required to monitor for ocular side effects. Serial ECGs done at subsequent office visits showed normal sinus rhythm with probable right ventricular hypertrophy, but no ventricular pre-excitation pattern was present as early as at her two-month-old visit. The patient continues to do well with no major illnesses or hospitalizations. She remains on propranolol 3 mg/kg/day. SVT training was reinforced with emphasis on prompt medical attention if the HR is 220 bpm or above and sustained.

## Discussion

The etiology of WPW comes from how the accessory pathway was formed during cardiac embryogenesis, specifically during the failure to resorb the myocardial syncytium at the annulus fibrosis of the atrioventricular valves. This results in faster electrical impulses seen as shorter PR intervals on ECG. These accessory pathways then have varying electrical conducting characteristics that may affect the speed of conduction, direction of conduction, and the refractory period. The location and number of pathways then affect the initiation or transmission of the arrhythmia, leading to WPW syndrome [4]. In about 3.4% of cases, there is a familial inheritance pattern in first-degree relatives with pre-excitation syndrome associated with WPW. There are genes implicated in the development of WPW. Examples include mutations of the protein kinase AMP-activated non-catalytic subunit gamma 2 (PRKAG2) gene, which can lead to cardiac glycogen overload, and the MYL2 gene, which encodes the regulator light chain associated with cardiac myosin beta heavy chain involved in conduction defects and eventual hypertrophic cardiomyopathy [4,5]. First-degree relatives may consider cardiac and genetic evaluation as well in the context of increased risk, given the rarity of WPW. Notably, most people with WPW pattern will never develop arrhythmia and remain asymptomatic [4]. This fact alone makes early and effective screening difficult.

A thorough clinical evaluation is essential, which should have a detailed history of present illness (including prenatal care, birth history, cardiac risk factors in the family, any infectious exposure), vital signs, and physical examination. The normal HR range for a newborn can range from 100 to 180 bpm. One case report highlights the importance of prenatal diagnosis, where SVT can cause heart failure leading to hydrops fetalis and eventual stillbirth if persistent for more than 12 hours or at rates of more than 230 bpm [6,7]. The diagnosis of WPW was confirmed postnatally on ECG [6]. On ECG, the hallmark WPW pattern consists of a short PR interval (<120 ms), a prolonged QRS complex (>120 ms), and QRS morphology of a slurred delta wave caused by ventricular pre-excitation. The absence of this pattern does not rule out the presence of other accessory pathways [4]. This patient did not have any cardiac abnormalities on fetal anatomy ultrasonography. Prompt diagnosis of SVT was challenging because the episodes were non-sustained in the outpatient setting. This patient had nonspecific symptoms of poor feeding and weight loss, but she remained normotensive and afebrile. The differential diagnosis is broad for nonspecific symptoms, including sepsis, dehydration, reflux, metabolic causes, cardiac, thyroid, etc. Sepsis and dehydration should always be ruled out. Studies to consider include chest radiography, echocardiography, complete blood count, basic metabolic panel, cardiac enzymes, B-type natriuretic peptide, thyroid function, and venous or arterial blood gas [4]. Unfortunately, SVT could be overlooked and go unrecognized until signs of cardiac failure are present, as seen in this patient’s echocardiogram. Sometimes the WPW pre-excitation is only seen transiently as well [7]. Cardiac instability is unlikely if the HR is less than 220 bpm in infants [6]. The parents were thoroughly educated on close monitoring of the HR to be less than this rate, and when to seek medical attention.

SVT management in children younger than one year old is by medical therapy rather than ablation. The first line of non-pharmacologic treatment is vagal maneuvers (e.g., ice pack to the forehead, immersion of the patient’s face in iced water, and the Valsalva maneuver). If this is unsuccessful, the next step is intravenous adenosine (0.05-0.3 mg/kg) for infants younger than one year old to slow electrical conduction and break the re-entry tachycardia back to sinus rhythm [7,8]. Escalation of care would include employing other antiarrhythmic agents such as amiodarone and propranolol along with electrical cardioversion [6]. Digoxin was formerly used for neonatal SVT, but can only be used in the absence of WPW syndrome due to the risks of ventricular fibrillation and the safety of electrical cardioversion [7]. If there is severe hemodynamic instability or if VT is suspected, prompt cardioversion (0.5 to 1 W sec/kg) is appropriate, as seen with this patient [8]. Chronic management strategies depend on timing and clinical severity of the initial SVT presentation [9]. Infants with SVT may remain on antiarrhythmics for the first months of life, but usually do not require them beyond the first year of life [6-8]. Prophylactic pharmacologic treatment for this age group is recommended to prevent symptomatic tachyarrhythmias at least until the end of the first year. This patient was weaned off oral amiodarone successfully and remained on propranolol. If SVT persists beyond one year old, spontaneous resolution is less likely and may require ablation therapy. Infants usually do not require ablation therapy; elective ablation can be performed in infants who weigh at least 15 kg if needed, but long-term complications for the developing myocardium and potential coronary artery stenosis are unknown [7]. There is a late recurrence risk of about 30% for SVT seen during the teenage years for those who had SVT as infants. This patient should be re-evaluated at that future time.

PCPs and Peds EM play a critical role in the early detection of SVT and WPW. Providers should maintain awareness of cardiac causes, especially for infants with persistent and nonspecific symptoms accompanied by tachycardia, poor perfusion, or feeding difficulties. The episodic nature of SVT and WPW adds to the diagnostic challenge. Early recognition and intervention can prevent acute heart failure and other morbidity and mortality. This patient regained weight appropriately with a modest weight loss at the time of SVT diagnosis. The patient was normotensive and afebrile with HR within the range for neonates. It is difficult to distinguish early SVT from common neonatal issues, especially since there was no sustained tachycardia above 220 bpm that would indicate clinical instability. Given the nonspecific symptoms and concerns of further weight loss and possible failure to thrive, further investigation with labs, ECG, or subspecialty consult remains available. ECG is a low-cost and non-invasive diagnostic tool. While a normal ECG does not rule out paroxysmal arrhythmias, ambulatory cardiac monitoring may be considered if clinical suspicion remains [10,11]. It is difficult to say if this approach would have changed this patient’s clinical course significantly, as it was the tachycardia and aberrant rhythm on ECG that were the most revealing only when the patient was symptomatic. There are no concrete guidelines on when to employ ECG screening when patients are asymptomatic. For asymptomatic patients with WPW pattern, the recommendations for further evaluation, risk stratification, electrophysiologic study, or accessory pathway ablation depend on age, risk factors, symptom history, comorbidities, baseline ECG pattern, and expert opinion. In general, asymptomatic young and healthy patients (no comorbid conditions, no risk factors) who have a WPW pattern are likely safe for watchful waiting with primary care and cardiology follow-up. If there are concerns for developing arrhythmia (e.g., clinical course or family history), patients should be referred to cardiology for discussion of risk stratification testing and/or EP study with accessory pathway mapping or ablation [4].

## Conclusions

In conclusion, this case underscores the importance of timely diagnosis and intervention for early-onset SVT for infants, especially in the outpatient setting. Cardiac etiology of symptoms should always be on the differential diagnosis for neonates. Given the potential subtle presentation of SVT, consider obtaining an ECG in neonates with persistent or intermittent tachycardia, poor feeding, unexplained weight loss, or signs of poor perfusion. SVT may present without fever or prior cardiac risk factors. There are no strict criteria for obtaining a screening ECG, but it is a low-cost tool with high-yield results. In addition, a normal ECG does not exclude intermittent arrhythmia, and early cardiology referral is a reasonable option. Despite the challenges in prompt diagnosis, this patient is currently doing well on the proper antiarrhythmic regimen. Awareness and education to expand the differential diagnosis to include cardiac etiology will improve clinical acumen and patient outcomes.
